# Insulin like growth factor – insulin like growth factor binding protein family in osteoporosis: mechanistic and clinical insights

**DOI:** 10.1080/07853890.2026.2665524

**Published:** 2026-05-05

**Authors:** Xiuwen Wang, Jie Zhang, Zhidan Luo

**Affiliations:** Department of Geriatrics, Chongqing Academy of Medical Sciences, Chongqing General Hospital, Chongqing University, Chongqing, China

**Keywords:** Bone metabolism, osteoporosis, IGFBPs, IGF, BMSCs

## Abstract

**Introduction:**

The imbalance between osteoblasts and osteoclasts is central to osteoporosis.Insulin like growth factor (IGF) has protective effects on bone. As an IGF-binding protein, the role of IGFBP in bone has also begun to come into the public’s view. IGFBP not only serves as an IGF reservoir but also acts independently, interacting with other ligands to regulate cell proliferation and differentiation. Due to the complexity of the molecular network of IGF-IGFBP, the specific mechanism by which IGF-IGFBP affects bone is still unknown. This review introduces the IGF-IGFBP network and its roles in osteogenesis and osteoclast differentiation, and explores its potential as a novel therapeutic target for osteoporosis.

**Method:**

Relevant studies were identified in PubMed and Web of Science using the keywords ‘IGFBP’, ‘Bone’, and ‘Osteoporosis’, followed by a comprehensive review of the literature.

**Results:**

IGF-IGFBP regulates bone metabolism by modulating the differentiation of bone marrow mesenchymal stem cells (BMSCs) into osteogenic and adipogenic lineages, as well as the differentiation process of osteoclasts. Current studies mainly focuson osteogenic differentiation, while research on adipogenic and osteoclastogenic differentiationis limited. Some IGFBP molecules show inhibitory effects in osteogenesis, but their detailed molecular mechanisms are not fully elucidated.

**Conclusion:**

The IGF-IGFBP family influences bone metabolism through multiple biological pathways. However, the precise molecular mechanisms by which this system regulates bone metabolism remain incompletely understood. Future studies are needed to systematically elucidate its functional networks and pathophysiological significance in bone metabolism.

## 1. Introduction

Osteoporosis is a common systemic bone disease characterized by decreased bone mass and destruction of bone microarchitecture [1]. Patients with osteoporosis have increased bone fragility and are more prone to fractures. The occurrence of fractures may cause a number of problems for patients, including disability, chronic pain and reduced quality of life, and the continuous investigation of the pathogenesis of osteoporosis and measures to prevent and control it has become an important endeavor and challenge for clinical and basic research. Several lines of evidence suggest that the IGF system is a mediator of postmenopausal osteoporosis. Serum IGF1 levels decline with age and are associated with age-related bone loss [2], and there is also a correlation between serum IGF1 levels and bone mass or the occurrence of osteoporotic fractures [3,4]. Studies have shown that the IGF system plays an important role in the regulation of bone metabolism. IGF stimulates osteoblast proliferation [5], enhances osteoclast differentiation [6], promotes bone matrix deposition [7,8], and delays osteoclast apoptosis [9,10]. In addition, IGF and its binding protein (IGFBP) are released through bone resorption and act as local determinants of site-specific coupling to bone formation [11]. Although the central role of IGF in bone metabolism is well established, in-depth investigation of the insulin-like growth factor-binding protein (IGFBP) family is emerging as a new entry point for unraveling the complex mechanisms of osteoporosis. IGF activity is regulated by at least six IGF-binding proteins (known as IGFBP1 through IGFBP6) [5]. The rationale for focusing on IGFBPs, rather than the broader IGF axis, lies in the fact that IGFBPs not only modulate IGF bioavailability through IGF-dependent pathways but, more importantly, also possess IGF-independent functions, directly interacting with cell surface receptors or extracellular matrix components to independently regulate osteoblast and osteoclast differentiation and function [12]. However, the precise functions of individual IGFBPs remain unclear, with the specific roles of IGFBP2, IGFBP3, and IGFBP4 in bone metabolism being particularly complex and controversial. Some members appear to act primarily as carrier proteins, prolonging the half-life of IGFs, while others are thought to exert dual effects in an autocrine/paracrine manner—either inhibiting IGF action by blocking their binding to receptors or potentiating IGF effects by providing a stable, available source of IGFs. This functional diversity suggests that the regulatory network of IGFBPs in bone homeostasis may involve broader pathophysiological processes. Notably, recent studies have indicated that members of the IGFBP family are closely associated with the regulation of inflammation. The pathogenesis of osteoporosis is also inextricably linked to inflammatory states: oxidative stress and inflammatory responses contribute to bone metabolic imbalance by modulating osteoblast and osteoclast activity [13]; the systemic immune-inflammation index is positively correlated with the risk of osteoporosis in middle-aged and elderly populations [14]; and targeting chronic inflammation has been proposed as a potential adjunctive therapeutic strategy for osteoporosis [15]. These findings suggest that inflammation may serve as a crucial bridging factor connecting aberrant IGFBP expression with bone metabolic imbalance, and that IGFBPs might indirectly influence bone remodeling processes by regulating local or systemic inflammatory responses. The complexity of the IGF-IGFBP system poses a challenge in the study of its biological function and its mechanism in osteoporosis. This review details the role of IGF-IGFBPs in osteoporosis, with a particular focus on the IGFBPs themselves, helping to deepen our understanding of osteoporotic pathogenesis and exploring the possibility of IGF-IGFBPs as potential therapeutic targets for osteoporosis.

## 2. IGF-IGFBP family

IGF1 and IGF2 are synthesized and secreted by the liver and bone, as well as by other organs [16]. They are members of the insulin superfamily of growth-promoting peptides, and are among the most abundant and ubiquitous peptide growth factors [16,17]. They share with insulin a complex system of heterotetrameric receptors and are associated signaling molecules, including the IGF1R, insulin receptor (IR) subtypes and their various hybrids [16]. In addition to acting locally in an autocrine or paracrine manner, IGF1 and IGF2 enter the bloodstream and interact with six high-affinity IGFBPs, which are the most important difference from insulin. IGFBP adds considerable complexity to the regulation of IGF bioavailability and signaling by modulating the tissue distribution of IGF and its binding to cellular receptors. The six mammalian proteins encoded by IGFBP1-IGFBP6 have been grouped into a superfamily [18] also including other growth-regulating proteins such as prostacyclin-stimulating factor (PSF), connective tissue growth factor (CTGF), nephroblastoma over-expressed gene (NOV) and cellular communication network factor 1 (CCN1). CTGF, NOV, and CCN1 are also collectively referred to as CCN proteins [19]. Although PSF has confusingly retained the gene name IGFBP7 and the other three proteins are still occasionally referred as IGFBP8, IGFBP9, and IGFBP10, respectively, or as IGFBP-related proteins (IGFBP-rPs), there is now broad consensus that only IGFBP1 through IGFBP6 should be designated as IGFBPs, based on their conserved protein structure and their high binding affinity for IGF1 and IGF2, which is a property that no other protein possesses[16]. The high binding affinity between IGFs and IGFBPs is largely attributed to the slow dissociation rate [20], making these complexes very stable once formed. While proteolytic hydrolysis of IGFBPs by enzymes including matrix metalloproteinases, deintegrin and metalloproteinase 28 (ADAM28) and plasma protein A (PAPPA) can reduce binding affinity to increase IGF bioavailability [21–24]. The mature IGFBP sequence, with 213–289 residues after signal peptide cleavage, has been described as consisting of three structural domains: disulfide-constrained amino-terminal and carboxyl-terminal structural domains, which are highly structurally conserved among the six proteins and between species, and a weakly structured central or linker structural domain, which is distinct among all family members [25]. IGFBP as a secreted protein, is also found intracellularly and interacts with many ligands other than IGF. Many of the cellular consequences of these interactions are thought to occur in the absence of regulation of the IGF-IGFR signaling pathway, and they are referred as IGF independent functions.

## 3. The relationship between osteoporosis and IGF-IGFBP family

**Figure 1.**
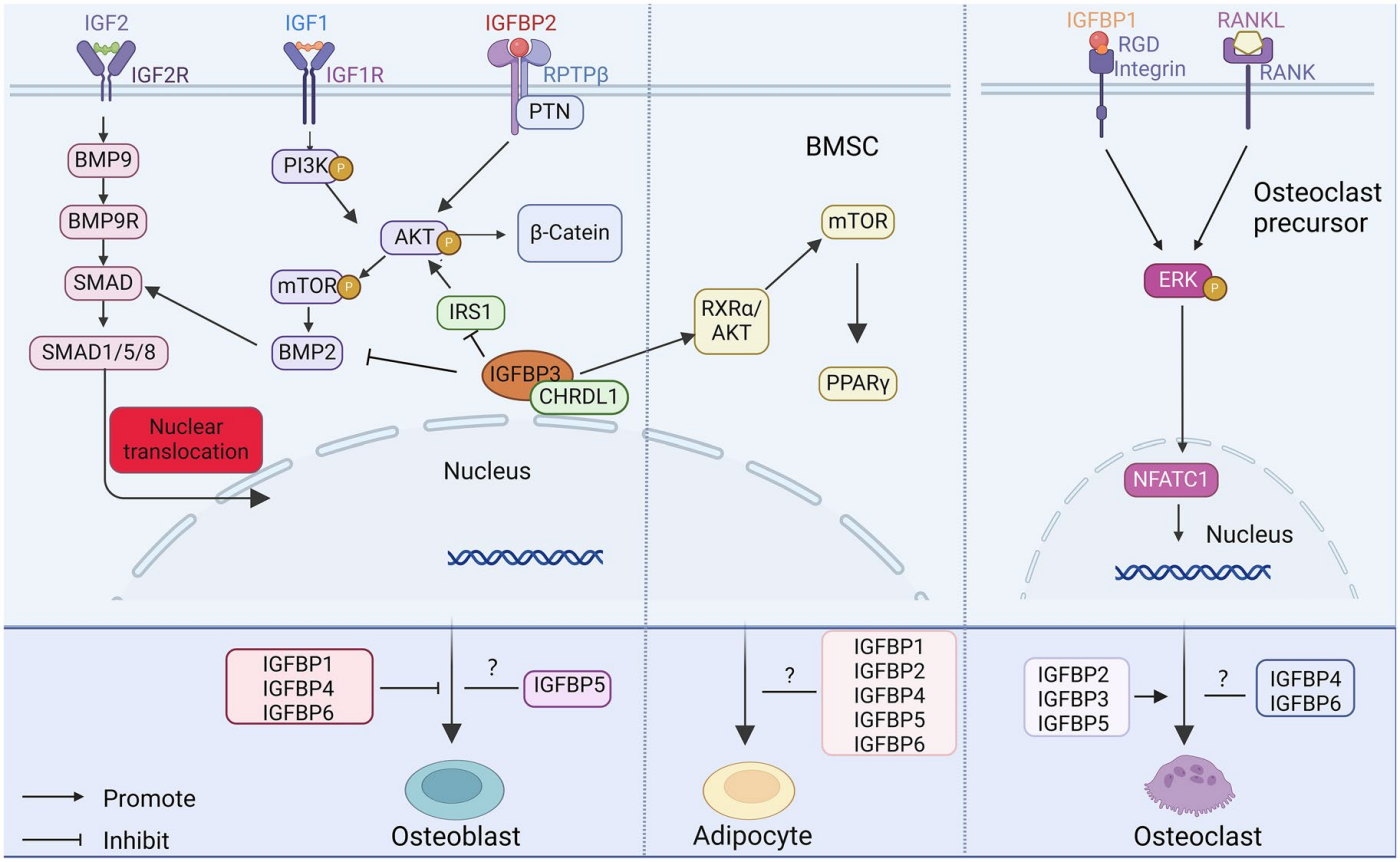
Effects of IGF-IGFBPs on bone. IGF1, IGF2 and IGFBP2 promote BMSC differentiation into osteoblasts, IGFBP1, IGFBP3, IGFBP4 and IGFBP6 inhibit BMSC differentiation into osteoblasts, and the effect of IGFBP5 on BMSC osteogenesis is unclear. Specifically, IGF1 promotes osteogenesis by stimulating BMP2 expression in BMSC through activation of the PI3/Akt/mTOR pathway; IGF2 promotes SMAD1/5/8 nuclear translocation and thus BMSC differentiation to osteoblasts through activation of the BMP9/SAMD pathway; IGFBP2 promotes BMSC osteogenesis by binding to the receptor RPTPβ and its other ligand PTN to facilitate the AKT/β-catein pathway; and CHRDL1 stabilizes intracellular IGFBP3 concentration to inhibit the IRS1/AKT/mTOR pathway and BMP pathway to inhibit BMSC osteogenesis. The mechanism by which IGFBP1, IGFBP4, and IGFBP6 inhibit the osteogenesis of BMSC is not clear. Studies related to the effects of IGFBP1, IGFBP2, IGFBP4, IGFBP5, and IGFBP6 on BMSC differentiation into adipocytes are lacking. IGFBP3 promotes BMSC differentiation into adipocytes through activation of the RXRα/AKT/mTOR/PPARγ pathway. IGFBP1 enters into osteoclastogenic precursor cells through its RGD domain and binds to its receptor integrin, which synergistically stimulates the phosphorylation of ERK in conjunction with RANKL, and further IGFBP1 activates NFATC1, which promotes osteoclast precursor cells to differentiate into osteoclasts. IGBP2, IGFBP3 and IGFBP5 promote osteoclast differentiation, but the specific mechanism is unknown. Research data on the effects of IGFBP4 and IGFBP6 on osteoclast differentiation is insufficient.

Osteoporosis is a common chronic disease in the middle-age and elderly population and is a skeletal disorder in which bone strength decreases leading to an increased risk of fracture, which is mainly characterized by low bone mineral density (BMD) and bone mass [26]. Bone is in a process of constant activity throughout life, and this activity is caused by bone formation by osteoblasts and bone resorption by osteoclasts so that the old bone is constantly replaced by new tissue. In the normal human body, bone formation and bone resorption are in balance so as to maintain the amount of bone mass, a phenomenon that Frost defines as bone remodeling [27]. The correct coupling of bone formation and resorption is required in order to achieve normal physiologic bone remodeling. Osteoblasts are derived from bone mesenchymal stem cells (BMSCs) in the bone marrow stroma and are responsible for bone matrix synthesis and its subsequent mineralization. BMSCs can differentiate into osteoblasts and adipocytes, and it is often assumed that these two directions of differentiation are in competition. Osteoclasts are multinucleated giant cells derived from mononuclear progenitor cells and are responsible for bone resorption. Any factor affecting osteoblasts and osteoclasts can influence the bone remodeling process. IGF-IGFBP system has been found to play an important role in regulating bone metabolism in existing studies (Figure 1).

### 3.1. The role of IGF-IGFBP in osteogenesis

IGF1 is the most abundant growth factor in the bone matrix and is essential for maintaining bone mass in adults. The concentration of IGF1 in bone marrow decreases in aging osteoporotic population and the concentration of IGF1 is lower in the bone matrix and bone marrow of aged rats than in young rats similarly [28]. In serum, the majority (about 80%) of IGF is present in a 150 kDa complex consisting of IGF, IGFBP3 and acid destabilizing subunit (ALS). A smaller proportion (about 20%) of IGF is associated with other serum IGFBPs in the 50 kDa complex, and less than 5% of IGF is present in the free form at 7.5 kDa. ALS is found primarily in serum and is a protein that binds to the IGF-IGFBP3 binary complex. This complex prolongs the half-life of serum IGF and promotes their endocrine actions. The ternary IGF/IGFBP3/ALS complex is thought to have difficulty crossing the capillary barrier compared to other IGF/IGFBP complexes [29,30]. It has been shown that liver IGF1 defects (LID) mice and ALS knockout (KO) mice exhibit relatively normal growth and development, despite a 75% and 65% reduction in serum IGF1 levels, respectively. In contrast, double-disrupted mice were generated by crossing with LID+ALS KO mice. These mice exhibited further reductions in serum IGF1 levels and significant reductions in linear growth. The LID+ALS KO mice had smaller total heights of proximal tibial growth plates as well as chondrocyte proliferation zones and hypertrophic zones. These mice also had a 10% decrease in BMD and more than a 35% decrease in periosteal circumference and cortical thickness. 4 weeks of IGF1 treatment restored the total height of the proximal tibial growth plate, suggesting that a threshold concentration of circulating IGF1 was necessary for normal bone growth [31]. The concomitant disruption of hepatic IGF1 and ALS genes in LID+ALS KO mice led to a further reduction in serum IGF1 and IGFBP3 levels mediated by a combination of insufficient hepatic IGF1 production and a decrease in the stabilization of circulating IGF1 derived from non-hepatic sources as a result of the disruption of the ALS gene. The absence of protective ALS proteins led to proteolytic cleavage of IGFBP3. This in turn impaired the ability of IGFBP3 to protect IGF1 because the affinity of fragmented IGFBP3 for IGF1 was reduced by 95% compared with the affinity of the intact protein [32]. In aged rats, IGF1 only or IGF1 plus IGFBP3 injections increased bone volume and bone density. However, bone formation in the IGF1only group occurred in clusters with structural defects in natural bone trabeculae, whereas injections of IGF1 and IGFBP3 improved bone volume and microarchitecture [28]. Selective stimulation of bone formation by recombinant human (rh) IGF in dystrophic and low bone conversion states [33]. These data suggest that IGF1, IGFBP3, and ALS play important roles in the pathophysiology of osteoporosis. Specifically, IGF1 can affect bone metabolism through multiple signaling pathways. *In vitro*, IGF1 promoted stem cell antigen 1 mesenchymal stem cell (Sca-1 MSC) osteogenic differentiation. IGF1 stimulated phosphorylation of IGF1R, insulin receptor substrate 1 (IRS1), PI3K, AKT and mTOR in Sca-1 MSC. Whereas, the addition of the inhibitor of mTOR, rapamycin (20 nM), or the PI3K inhibitor, LY294002 (10 μM), to the culture medium concomitantly with IGF1 intervention impaired mineralization but did not affect cell growth and survival. While the PI3K inhibitor decreased the phosphorylation of PI3K, AKT, and mTOR, rapamycin inhibited only the phosphorylation of mTOR, suggesting that IGF1 in Sca-1 MSCs activated mTOR through the PI3K-AKT pathway and induced its differentiation toward osteoblast [28]. Additional experiments showed that the intact IGF1 induced PI3K-AKT signaling cascade was essential for bone morphogenetic protein 2 (BMP2), and the BMP-SMAD signaling pathway is a key pathway regulating the differentiation and maturation of BMSCs into osteoblasts [34,35]. Manipulation of this pathway could promote bone remodeling and fracture repair [36]. Similarly, IGF2 was recognized as a potent molecule for promoting osteogenesis, and IGF2 expression enhanced BMP9 induced early osteogenic marker alkaline phosphatase (ALP) activity and expression of late markers. IGF2 has been shown to enhance BMP9 induced ectopic bone formation in stem cell implantation assays. In a perinatal limb explant culture assay, IGF2 enhanced BMP9 induced endochondral ossification, whereas IGF2 itself promoted the expansion of hypertrophic chondrocyte zones in cultured limb explants. Mechanistically, IGF2 was further shown to enhance BMP9 induced BMPR-SMAD reporter gene activity and SMAD1/5/8 nuclear translocation. The PI3K inhibitor LY294002 eliminated the potentiating effect of IGF2 on BMP9 mediated osteogenic signaling, and could directly inhibit BMP9 activity. These results suggest that BMP9 cross-talks with IGF2 during MSC osteogenic differentiation *via* the PI3K/AKT signaling pathway [37].

A population based cross-sectional study of BMD measurements at the left hip, lumbar spine, and heel, as well as risk markers for osteoporosis, was conducted in 350 elderly women (the mean age was 73 years). BMD values were significantly negatively correlated with IGFBP1 values at each site of measurement, and significantly positively correlated with IGF1 values at all sites except the lumbar spine [38]. Another cross-sectional study of serum IGFBP1 levels and BMD in 1139 community-dwelling men and postmenopausal women (estrogen-free) aged 44–98 years found that in both sexes, IGFBP1 levels increased linearly with age and decreased with body mass index (BMI) quartiles. After adjusting for age and BMI, there was no significant association between IGFBP1 and BMD of the hip or spine. IGF1 and IGFBP1 were weakly negatively correlated with each other. These findings suggest that if IGFBP1 plays an important role in bone metabolism, it may be mediated or confounded by weight [39]. Another clinical study found a positive linear relationship between IGFBP1 and severe osteoporotic fracture (including hip, spine, shoulder, and wrist fractures). The relationship between IGFBP1 and fracture risk was not confounded by IGF1 or BMI. However, femoral neck BMD mediated 56% of the total ‘effect’ of IGFBP1 on hip fracture risk [40]. This implies that IGFBP1 may be an important factor in bone turnover and that further studies are needed to investigate the relationship between the inhibitory component of IGF1 and bone loss. BMSCs were inoculated on cell-free bone blocks from young and old donors undergoing osteoarthritis-related hip surgery in an experiment. BMSCs cultured in conditioned medium from old donor bone showed higher osteogenic gene expression and ALP activity than BMSCs exposed to conditioned medium from young donor bone. ELISA and Luminex analysis of the conditioned media showed that the levels of bioactive factors were similar between age groups; however, IGFBP1 concentrations were significantly higher in the young donor samples [41]. The results of this experiment suggest that high concentrations of IGFBP1 may inhibit BMSCs osteogenic differentiation. Current research on the specific mechanisms by which IGFBP1 directly influences osteogenic differentiation remains insufficient. However, studies have demonstrated that IGFBP1 can activate the MAPK-ERK signaling pathway in lung adenocarcinoma cells, thereby promoting tumor proliferation, invasion, and migration [42]. The MAPK-ERK signaling pathway is precisely one of the key pathways influencing osteogenic differentiation. Whether IGFBP1 similarly regulates osteogenic differentiation by affecting this pathway warrants further investigation.

In cellular experiments, IGFBP2 was found to enhance the osteogenic capacity and osteogenic gene expression of human mesenchymal stem cells (hMSCs) [43]. Knockdown of IGFBP2 resulted in a significant delay in the differentiation of MC3T3 osteoblasts and a decrease in osteocalcin expression. Primary cranial osteoblasts from IGFBP2 KO mice had reduced osteogenic capacity, and addition of IGFBP2 rescued the differentiation program. Whereas overexpression of IGFBP2 accelerated the differentiation process and increased the total number of differentiated cells [44]. *In vivo*, the effects of IGFBP2 on bone were found to be sex-dependent, with male IGFBP2 KO mice having significantly reduced bone mass and administration of a peptide containing the receptor-binding domain of IGFBP2 stimulating bone formation in these animals. Female IGFBP2 KO mice showed no significant change in bone mass but lost more bone mass after ovariectomy (OVX) than OVX wild-type mice. This suggests that female mice require IGFBP2 to maintain bone mass in the absence of estrogen [45]. IGFBP2 could stimulate osteoblast differentiation, and the trophic effects of IGFBP2 in bone were mediated through its binding to receptor tyrosine phosphatase β (RPTPβ). Another important ligand of RPTPβ was the pro-telephysial nephrocyte (PTN), which also stimulated osteoblast differentiation. Changes in PTN and RPTPβ in whole bone of IGFBP2 KO mice were determined and found that the expression of PTN and RPTPβ was increased in females compared to males. Knockdown of PTN in osteoblasts inhibited differentiation and was rescued by addition of PTN to the incubation medium. Estradiol-stimulated PTN secretion and PTN knockdown blocked estradiol stimulated osteoblast differentiation. Addition of PTN to the culture medium of IGFBP2-silenced osteoblasts stimulated osteogenesis, whereas anti-fibronectin-3 antibody inhibited this response by inhibiting the binding of PTN to RPTPβ. Estrogen stimulated PTN secretion and downstream signaling in IGFBP2-silenced osteoblasts, and these effects were inhibited by antifibrillin-3. Thus absence of IGFBP2 expression was accompanied by a compensatory up-regulation of PTN and RPTPβ expression in osteoblasts, which was greater in female mice due to estrogen secretion, and this compensatory change might partially account for the maintenance of normal bone mass in female mice [46]. Administration of heparin-binding domain (HBD) peptide to IGFBP2 KO mice increased bone formation, inhibited bone marrow adipogenesis, restored trabecular bone mass, and reduced bone resorption. Skeletal salvage in IGFBP2 KO mice was characterized by decreased phosphatase and tensin homolog (PTEN) expression followed by enhanced AKT phosphorylation in response to IGF1 and increased β-catenin signaling through stimulation of its cytoplasmic accumulation and phosphorylation of serine 552. That is, the HBD peptide of IGFBP2 promoted osteogenesis by activating the IGF1/AKT and β-catenin signaling pathways [47]. In contrast, as found in IGFBP2 transgenic mice, the transgenic mice had significantly reduced bone size and bone mineral content. Interestingly, elevated levels of IGFBP2 did not result in reduced bone density, suggesting that IGFBP2 negatively affects bone size and mineral content but not bone maintenance in adult mice [48]. Although most studies have concluded that IGFBP2 has beneficial effects on bone, a small number of studies have concluded that IGFBP2 has detrimental effects on bone, and in one clinical study it was found that the observed increase in circulating levels of IGFBP2 with advancing age was associated with deleterious effects on BMD in both men and women [49]. Serum IGFBP2 was elevated in patients after bariatric surgery, which might improve glycemia and promote bone resorption and inhibit the effects of IGF1. Trabecular bone loss and cortical bone loss were reduced in IGFBP2 KO mice after 4 weeks of vertical sleeve gastrectomy (VSG) [50]. These data suggest a possible adverse effect of IGFBP2 on bone in the context of weight loss.

IGFBP3 has been commonly thought to act as a stabilizer and transporter protein for IGF1, and it has recently been found that IGFBP3 can play a role in bone maintenance in an IGF1-independent pathway. Examination of BMD, osteoblast proliferation in transgenic mice overexpressing IGFBP3 revealed that IGFBP3 overexpression impaired osteoblast proliferation and had significant negative effects on bone formation [51]. Intervention of BMSCs osteogenesis with the addition of 50 ng/ml of IGFBP3 to the culture medium revealed that IGFBP3 inhibited osteoblast differentiation by suppressing BMP-2 signaling induced activity of the smad-binding element (SBE) reporter [52]. In another experiment investigating whether hypoxic culture conditions (2% O_2_) inhibited spontaneous mineralization and osteogenic differentiation of human adipose stem cells (ASCs), hypoxia inhibited spontaneous mineralization and osteogenic differentiation of ASCs and up-regulated the mRNA and protein expression of IGFBPs in ASCs. Although treatment with rhIGFBP did not affect ASC osteogenic differentiation, siRNA-mediated inhibition of IGFBP3 attenuated hypoxia-inhibited ASC osteogenic differentiation. In contrast, overexpression of IGFBP3 by lentiviral vectors inhibited ASC osteogenic differentiation and reduced ASC proliferation. This suggests that hypoxia inhibits spontaneous mineralization and osteogenic differentiation of ASC through intracellular IGFBP3 upregulation [53]. Chordin like-1 (CHRDL1) is an antagonist of BMP that inhibits osteogenic differentiation by binding to BMP and blocking its interaction with the BMP receptor. CHRDL1 and IGFBP3 were up-regulated in trabecular bone of aged mice. Mechanistic explorations suggested that CHRDL1 directly bound to IGFBP3 and attenuated the degradation of IGFBP3. CHRDL1 and IGFBP3 inhibited the mechanistic target of IRS/AKT/protein kinase complex mechanistic target of rapamycin complex 1 (mTORC1) signaling in osteogenic differentiated progenitor cells. Overexpression of IGFBP3 attenuated CHRDL1 silencing-induced enhancement of progenitor cell osteogenesis [54].

IGFBP4 and IGFBP5 are the main products of osteoblasts among all IGFBPs. IGFBP4 is of particular interest because its abundance is regulated by a number of bone-active substances, including parathyroid hormone (PTH) and 1,25-Dihydroxvitamin D3 (1,25(OH)2D3), and thus may be mechanistically involved in their actions in the skeleton [55]. IGFBP4 overexpression was targeted to osteoblasts in transgenic mice using the human osteocalcin promoter(OC-BP4 mice), which had been shown to specifically direct transgene expression in osteoblasts and osteocytes. The results showed a 25-fold increase in IGFBP4 protein levels in the skull of transgenic mice compared with endogenous levels. Interestingly, IGFBP5 levels were reduced in OC-BP4 mice, possibly because of compensatory alterations in IGF1 action. Morphometric measurements showed a reduction in femur length and total bone volume in transgenic animals compared with controls. Quantitative histomorphometric measurements of the distal femur showed that the bone conversion rate was significantly reduced in OC-BP4 mice. The osteoblast number, bone length and bone formation rate in OC-BP4 mice were approximately half that of control mice. At birth, OC-BP4 mice were normal in size and weight but exhibited marked postnatal growth retardation [56]. IGFBP4 is the most abundantly expressed IGFBP species at the mRNA and protein levels under basal and osteogenic conditions. Although there was no difference in IGFBP4 expression under osteogenic conditions, there was an increase in the expression and activity of pregnancy-associated plasma protein A (PAPPA—an IGFBP4 protease), which led to an increase in IGFBP4 protein hydrolysis in differentiated cell cultures. In addition, osteogenic conditions were characterized by increased expression of IGF2 (an activator of PAPPA) and decreased expression of stanniocalcin-2 (an inhibitor of PAPPA) [57]. This suggests that IGFBP4 may exert its inhibitory effect on osteogenesis through IGF-dependent mechanisms. Another study on fracture healing in male idiopathic osteoporosis demonstrated that CircRNA_000639/IGFBP4 regulates osteogenesis by modulating iron loading in BMSCs [58].

IGFBP5 is an osteoblast-secreted protein stored in bone that acts by incorporating into the mineralized bone matrix. Subcutaneous injection of rhIGFBP5(50 μg/kg mice) increased serum OC and bone ALP activity but did not significantly increase serum IGF1, suggesting that the effects of rhIGFBP5 on bone are not mediated by an increase in circulating IGF1. *In vitro* experiments demonstrated that rhIGFBP5 increased osteoblast differentiation capacity, ALP activity and OC levels in a dose-dependent manner [59]. Overexpression of IGBP5 in hMSC increased ALP staining and expression of osteogenic markers, whereas knockdown of the gene impaired osteogenesis. Overexpression of IGFBP5 also significantly enhanced extracellular signal-regulated kinase 1/2 (ERK1/2) phosphorylation, whereas knockdown of IGFBP5 inhibited this signaling activity [60]. Although IGFBP5 has been implicated as a bone-enhancing factor in several studies, conversely, it has also been shown to act as an inhibitor of bone formation. Trabecular bone volume was transiently reduced in IGFBP5 transgenic mice under the control of the OC promoter, secondary to a reduction in the number and thickness of bone trabeculae and a transient reduction in the rate of bone mineral attachment. Osteoblast numbers were normal, suggesting impaired osteoblast function. Stromal cells overexpressing the IGFBP5 transgene exhibited reduced expression of ALP, OC, core binding factor 1, and type I collagen transcripts compared with cells from wild-type animals [61]. MC3T3-E1 cells expressed ALP and OC mRNA at 1 week and 4 weeks after osteogenic induction. Overexpression of IGFBP5 delayed the timing of ALP and OC mRNA expression in MC3T3-E1 cells, decreased type I collagen and bone bridging protein mRNA and ALP activity and inhibited the formation of mineralized nodules. In conclusion, overexpression of IGFBP5 may reduce osteoblast function by binding IGF in the bone microenvironment [62]. The reasons for the opposite results are worth exploring and may be due to inconsistencies in IGFBP5 concentrations and differences in mouse models.

IGFBP6 inhibits differentiation of myoblasts and osteoblasts. IGFBP6 protein was found to be downregulated during the osteogenic differentiation of MC3T3-E1 cells. Overexpression of IGFBP6 in MC3T3-E1 and human osteoblasts inhibited nodule formation, osteocalcin mRNA expression, and ALP activity. Accumulation of IGFBP6 in the culture medium was not required for any of these effects, suggesting that IGFBP6 inhibited osteoblast differentiation through an intracellular mechanism [63]. Other experiments suggested that IGFBP6 could translocate to the nucleus to play a role. Addition of IGFBP6 significantly increased pluripotency-associated markers and muscle differentiation markers in placental mesenchymal stem cells (PMSCs) at early time points, but the latter decreased over time. On the other hand, silencing IGFBP6 decreased pluripotency and differentiation markers at early time points. Levels of these markers increased as IGFBP6 levels were restored [64]. In terms of mechanisms, IGFBP6 directly interacted with thyroid hormone receptor α1 (TRα1) *in vitro* and *in vivo*. IGFBP6 negatively regulated ligand activation of TRα1-induced growth hormone promoter activity and inhibited triiodothyronine (3,3′,5-triiodo-L-thyronine, T3) in osteoblasts induced osteocalcin mRNA transcription. When cells were transfected with IGFBP6 in the presence of T3, ALP activity in osteoblasts was significantly reduced. It was demonstrated that overexpression of IGFBP6 inhibited T3-TR axis regulated osteoblast differentiation [65]. A study on liver fibrosis revealed that IGFBP6 inhibits TGF-β/SMAD signaling to improve pulmonary fibrosis [66]. Whether IGFBP6 suppresses osteogenic differentiation through this pathway requires further investigation.

### 3.2. The role of IGF-IGFBP in osteoclastogenesis

IGFBP1 is primarily produced by hepatocytes. Besides acting as a key regulator of IGF1, it also functions as an endocrine factor in its own right. Recombinant IGFBP1 was found to enhance differentiation of isolated osteoclasts but was inhibited by an IGFBP1 blocking antibody. Intraperitoneal injection of IGFBP1 into WT mice led to bone loss. The expression of IGFBP1 and FGF21 in the liver iwas increased in OVX mice, and treatment with an anti-IGFBP1 antibody alleviated OVX-induced osteoporosis. Administering anti-IGFBP1 treatment in FGF21-Tg mice, which overexpress FGF21, was found to reduce bone loss in these mice. Mechanistic studies indicated that IGFBP1 acted through its RGD domain on the integrin β1 receptor on osteoclast precursors. This enhanced RANKL-stimulated Erk phosphorylation and activation of NFATc1, leading to increased osteoclast differentiation [67].

IGFBP2 increased with age and was a predictor of low BMD in older men and women; higher serum IGFBP2 predicted lower BMD and was associated with increased bone resorption markers independent of age, body weight and sex hormones [68]. IGFBP2 might be an important regulator of osteoclastogenesis, with its heparin-binding and IGF-binding domains being involved in the formation of fully differentiated and functional osteoclasts. Global deletion of the IGFBP2 gene resulted in inhibition of bone conversion, characterized by a concurrent reduction in both osteoblasts and osteoclasts. Culture of IGFBP2 KO bone marrow cells revealed reduced osteoclast numbers and impaired resorption. Addition of full-length IGFBP2 restored osteoclast differentiation, fusion and resorption [69].

In postmenopausal women, IGF1 and IGF1/IGFBP3 ratios were negatively correlated with CTX levels [70], and examination of bone resorption markers in transgenic mice overexpressing IGFBP3 had shown that overexpression of IGFBP3 increased osteoclast numbers and bone resorption [51]. Another study showed that IGFBP3 increased the viability of mouse bone marrow macrophages, but osteoclast differentiation of these cells was blocked by IGFBP3 in a dose-dependent manner [71]. IGFBP3 promoted bone resorption in mice but blocked osteoclast differentiation in cell culture. This discrepancy in outcomes may be attributed to differences in IGFBP3 dosage between *in vivo* and *in vitro* conditions, as well as the complexity of the *in vivo* environment, where the integrated regulation by multiple cell types leads to divergent results. For future in-depth investigation, it may be worthwhile to consider performing co-culture experiments with various bone cell types, or to simulate the *in vivo* microenvironment *in vitro* (e.g. by incorporating matrix components or applying fluid shear stress), in order to verify the effects of IGFBP3 on osteoclast differentiation under different contexts. Additionally, study of IGFBP3 in osteoclastogenesis is lacking and further experimental validation is needed.

IGFBP5 stimulated bone resorption *in vitro* by stimulating osteoclast formation in an IGF1-independent manner and by IGF1-dependent activation of mature osteoclasts. When IGFBP5-induced osteoclasts were co-cultured with MC3T3-G2 cells, the formation of resorption pits was observed, whereas no resorption pits were detected in IGFBP5-induced osteoclast-like cells cultured in the absence of MC3T3-G2 cells. This indicated that the differentiation of osteoclast precursor cells into osteoclasts induced by IGFBP5 required the presence of osteoblasts to acquire bone resorption activity [72].

Another experiment testing the effect of IGFBP5 on osteoclast activity and osteoclast formation found that IGFBP5 significantly stimulated pit formation by preexisting osteoclasts in normal culture medium, and its stimulatory effect was completely blocked by IGF1 antibody. However, IGFBP5 did not affect the bone resorption activity of isolated rabbit osteoblasts. When IGFBP5 was added to normal culture medium after denaturation of preexisting osteoclasts, IGFBP5 dose-dependently stimulated the formation of osteoclast-like cells regardless of the presence of IGF1 antibody [73].

### 3.3. The role of IGF-IGFBP in lipogenesis

CHRDL1 stabilized IGFBP3 binding to promote adipocyte differentiation. They activated AKT/mTORC1 signaling during adipogenic differentiation independently of IRS1. CHRDL1 enhanced the interaction between cytosolic IGFBP3 and retinoid X receptor-like α (RXRα) during adipogenesis. overexpression of IGFBP3 alleviated CHRDL1 silencing-induced attenuation of adipogenic differentiation [54]. A disintegrin and metalloprotease 12 (ADAM12) was associated with adipogenesis, and knockdown ADAM12 in 3T3-L1 cells reduced the number of cells in proliferating preadipocytes, delayed preadipocyte to adipocyte differentiation, and increased lipid accumulation in mature adipocytes, and PPARγ signaling was also downregulated by ADAM12 knockdown. The pathway most affected by ADAM12 knockdown was the regulation of IGF activity by IGFBP, and the IGF/mTOR signaling pathway was down-regulated in adipocytes. ADAM12 cleaved IGFBP3 and IGFBP5, and IGBP3 interacted with PPARγ to impede its regulation. ADAM12 regulated cell proliferation in preadipocytes through IGFBP/IGF/mTOR signaling and delayed differentiation by altering PPAR signaling, leading to lipid imbalance in mature adipocytes [74].

### 3.4. The role of IGF-IGFBP in osteogenesis and osteoclastogenesis coupling

Bone homeostasis is a dynamic equilibrium dependent on interactions between osteoblasts and osteoclasts. IR was found in osteoblasts and osteoclasts, whereas IRS was found only in osteocytes. IRS is a key factor in insulin and IGF1 signaling. It had a docking role with Src homology 2 signaling molecules in cells, and signals through Src homology 3 to phosphorylate downstream Ras molecules, thereby activating the ERK1/2 pathway, which regulated osteoblast growth, proliferation and differentiation [75,76]. Phosphorylation of IRS tyrosine residues activated PI3-K [76], which implied promotion of the conversion of phosphatidylinositol-4,5-bisphosphate to phosphatidylinositol-3,4,5-trisphosphate. Phosphatidylinositol-3,4,5-trisphosphate activated downstream AKT, which in turn regulated osteoblast proliferation, survival, and energy metabolism *via* mammalian targets of rapamycin, cell death BCL-2 antagonists, and forkhead box O1 [77,78]. Osteoblasts without IRS1 failed to produce receptor activators of NF-κB ligand/osteoclast differentiation factor, which was essential for osteoclast development. Bone resorption was found to be reduced in IR-deficient mice, but bone mass reduced in these mice, suggesting a stronger inhibition of bone formation than bone resorption [79].

GH and IGFBP5 can directly promote osteoclast differentiation and maturation and also stimulate bone resorption through localized IGF1/II production by osteoblasts [72]. IGFRI expression has been demonstrated on mature rabbit osteoblasts as well as human osteoclasts [80,81]. IGF1 enhanced the formation of osteoclast-like cells in long-term bone marrow cultures [82]. In contrast, IGF1 had an inhibitory effect on stimulated bone resorption in bone organ cultures [83]. It is believed that IGF1/II is incorporated into the bone matrix by binding to IGFBP5 and hydroxyapatite for release during osteoclastic bone resorption. Thus, IGF1 can act as one of several coupling agents by activating bone formation and bone resorption, and the amount of IGF1 released from the bone matrix should activate a proportional response of osteoblasts to produce sufficient osteoid to fill the resorption gap [84]. In mature osteoblasts, IGFBP5 was enhanced by Runx2 but was no longer able to stimulate c-Src activation because tyrosine kinases were downregulated at this stage. IGFBP5 produced by osteoblasts stimulated osteoclastogenesis and bone resorption, acting as an osteoblast-osteoclast coupling factor [85]. In addition, pro-inflammatory cytokines such as TNF-α, IL-1β and IL-6 promote osteoclastogenesis, and GH and IGF1 stimulate the production of these cytokines in osteoblasts [86,87]. In contrast, cytokines released in the bone matrix during bone resorption, such as TGF-β and IGF1, also affect osteoblast activity [88].

### 3.5. IGF-IGFBP interactions with other hormones affect bone metabolism

GH and IGF1 stimulated preadipocytes at different developmental stages and had been shown to have direct effects on osteoblasts [89]. IGF1 activity on osteoblasts was increased by the presence of GH and IGFBP3 [90]. IGF1 did not replace GH in promoting this differentiation, but its mitogenic action selectively promoted cell proliferation in young differentiated clones. Both GH and IGF1 had been recognized as potential anabolic agents with physiological roles in bone acquisition and maintenance [91]. Administration of GH and IGF1 was capable of stimulating longitudinal bone growth in animals and humans through direct action [57] and indirectly increasing local production of the IGF1 [92,93]. GH and IGF1 had separate and distinct functions [94], when the two compounds were given together they act synergistically [95].

Levels of GH, IGF1, and some IGFBP were reduced in older adults [96], and low circulating levels of IGF1 in osteoporotic subjects [4,97] and older women had been associated with greater femoral bone loss [3]. Interestingly, age-dependent attenuation of GH, IGF1 and IGFBP3 levels in healthy men was not associated with reduced BMD [98,99], suggesting other hormonal interactions. Androgens had been shown to inhibit osteoblast apoptosis and prolong osteoblast lifespan [100]. Various studies had shown that osteoblasts were directly stimulated by androgens. DHT, an androgen that was not converted in estradiol, stimulated mineralization and increased the androgen receptor through mechanisms distinct from 1,25 (OH)D3 and TGF-β. Osteoclasts expressed the IGF1 receptor, and IGF1 had a direct effect on their function [80]. *In vitro*, IGF1 induced the synthesis of RANKL and consequently osteoclastogenesis [101]. Induction of RANKL by IGF1 explained the stimulatory effects of IGF1 on bone resorption, whereas induction of osteoprotegerin by GH might moderate these effects [102].

**Figure 2.**
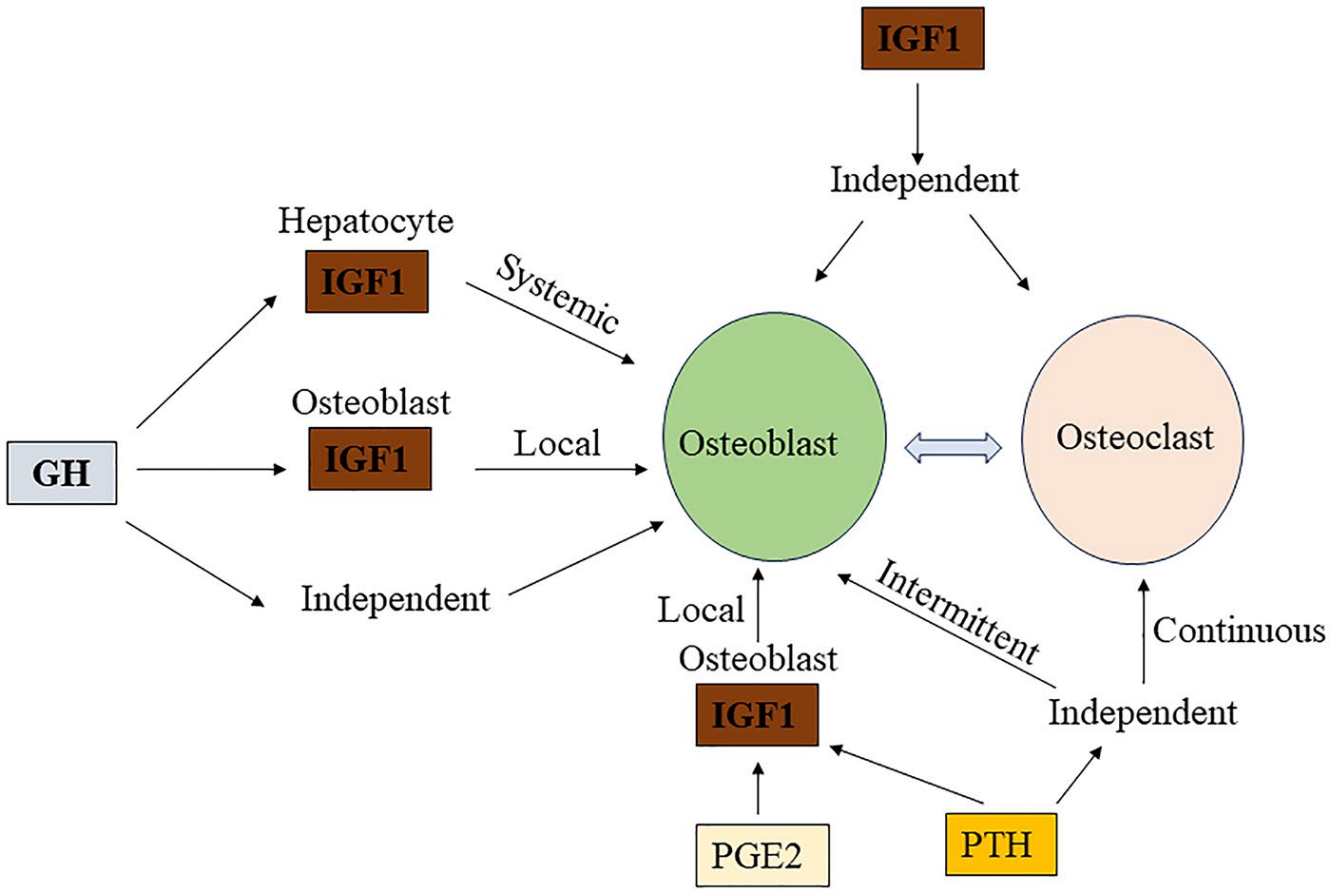
GH, PTH, PEG2 and IGF1 have synergistic effects on bone. GH stimulates the production of IGF1 in hepatocyte and osteoblasts. PTH and PEG2 promote the production of IGF1 in osteoblasts. GH and IGF1 promote osteoblast differentiation directly. Intermittent administration of PTH promotes osteoblast differentiation, while continuous administration of PTH promotes osteoclast differentiation. IGF1 can also promote osteoclast differentiation directly.

IGF1 was produced locally by osteoblasts and was controlled not only by GH but also by other factors [103]. It was well known that both PTH and prostaglandin E2 (PGE2) upregulated IGF1 mRNA levels in the Ob cell model and that cAMP acted as a second intracellular signaling agent [104]. Instantaneous treatment stimulated collagen synthesis and the stimulatory effect was mediated by locally produced IGF1 [105,106]. Intermittent PTH treatment enhanced osteoblast differentiation through an IGF independent mechanism, and continuous PTH treatment promoted osteoclastogenesis [107]. Different exposure times mediated different signal transduction systems with different effects on osteoblast differentiation *in vitro* [108] (Figure 2).

### 3.6. The interaction of IGF-IGFBP with inflammation in bone metabolism

**Figure 3.**
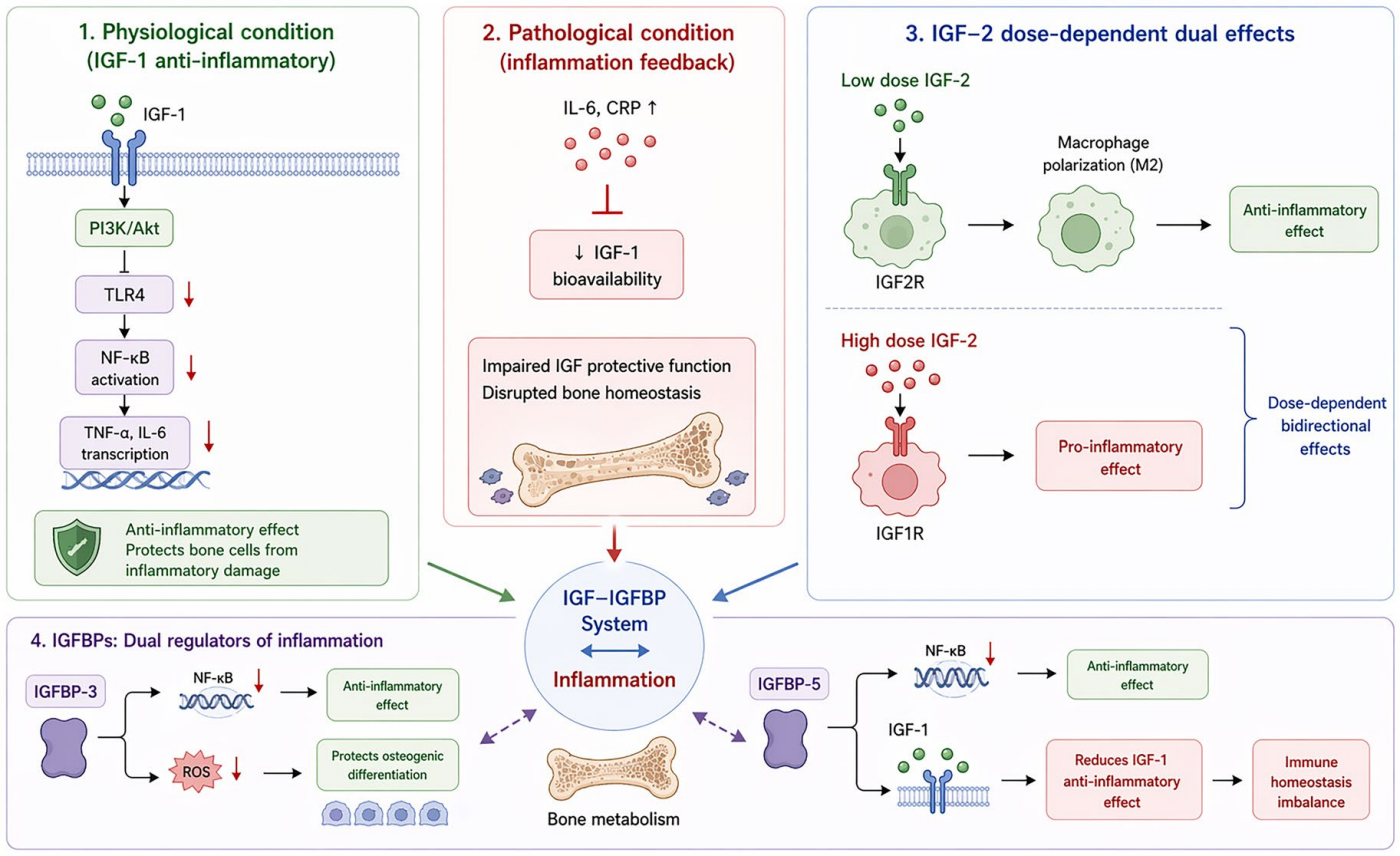
Simplified mechanism of the IGF-IGFBP-inflammation axis in bone homeostasis. Under physiological conditions, IGF-1 exerts anti-inflammatory effects by inhibiting TLR4 and NF-κB target genes (e.g. TNF-α, IL-6) through the PI3K/Akt pathway. Under pathological conditions, elevated inflammatory factors (e.g. IL-6, CRP) suppress IGF-1 bioavailability. IGF2 exhibits dose-dependent bidirectional effects: low doses induce macrophage polarization toward an anti-inflammatory phenotype *via* IGF2R, whereas high doses may exacerbate inflammation through IGF1R. The IGFBP family both regulates IGF bioavailability and independently modulates inflammation. IGFBP-3 exerts anti-inflammatory effects by inhibiting the NF-κB pathway and reduces oxidative stress to protect osteogenic differentiation. IGFBP-5 has dual effects: it inhibits NF-κB activation on one hand, but on the other hand, it binds to IGF-1 and attenuates its anti-inflammatory function, leading to immune imbalance.

IGF itself possesses bidirectional immunomodulatory functions and may serve as a key bridge connecting bone metabolism and inflammation. Under physiological conditions, IGF-1 inhibited the expression of Toll-like receptor 4 (TLR4) *via* the PI3K/Akt signaling pathway and reduced the transcription of NF-κB target genes (such as TNF-α and IL-6), thereby exerting anti-inflammatory effects and protecting skeletal cells from inflammatory damag [109]. Under pathological conditions (such as aging or sepsis), persistently elevated inflammatory factors (e.g. IL-6, CRP) could inhibit the bioavailability of IGF-1[110]. Interestingly, IGF2 exhibited dose-dependent bidirectional effects: low-dose IGF2 activated the IGF2 receptor (IGF2R), inducing macrophage polarization towarded an anti-inflammatory phenotype, whereas high-dose IGF2 might exacerbate inflammatory responses, potentially through IGF1R [110]. The IGFBP family not only acted as a regulator of IGF bioavailability but also functioned as an independent inflammatory modulator. Studies have found that IGFBP-3 can exert anti-inflammatory effects directly in tumor cells by inhibiting the NF-κB pathway [111] and can reduce oxidative stress and protect osteogenic differentiation in a methylglyoxal-induced bone loss model [112]. IGFBP-5 exhibited more complex dual regulation: on one hand, it bound to ANXA2 to inhibit NF-κB activation, exerting anti-inflammatory effects; on the other hand, it bound to IGF-1, thereby attenuating IGF-1’s anti-inflammatory function and leading to immune homeostasis imbalance [113]. IGFBP-6 was involved in regulating vascular inflammation by promoting immune cell chemotaxis [114]. The studies indicate a multi-dimensional interactive regulation between the IGF-IGFBP system and inflammatory factors: IGF may protect bone through anti-inflammatory pathways, while IGFBPs bidirectionally modulate immune responses through both IGF-dependent and IGF-independent mechanisms. Conversely, the inflammatory state can, in turn, remodel the expression and function of the IGF-IGFBP system. Future research should focus on this regulatory network to explore the feasibility of intervening in the ‘IGF-IGFBP-inflammation’ axis to restore bone metabolic balance. This could provide a novel therapeutic strategy for osteoporosis that offers both pro-osteogenic and anti-inflammatory effects (Figure 3).

## 4. Conclusion

Some clinical studies have found that the concentration of IGF-IGFBP in the blood is correlated with BMD. However, the results across various clinical studies are not entirely consistent. A study based on a UK population database revealed that IGF-1 and IGFBP-3 are associated with a reduced risk of osteoporosis. In contrast, no such association was found in the FinnGen cohort, suggesting that the relationship between IGF-IGFBP and osteoporosis may exhibit population-specific differences [115]. Future research should investigate these associations across diverse populations. Notably, current knowledge regarding the expression heterogeneity of IGFBPs within distinct bone compartments remains limited. The cellular origins, spatial distribution, and local functions of individual IGFBPs across different anatomical regions (e.g. trabecular versus cortical bone, endosteal versus periosteal surfaces) and bone marrow niches have yet to be systematically characterized. This lack of region-specific expression data hampers a comprehensive understanding of the complex roles of IGFBPs in bone metabolism. Future studies should employ single-cell RNA sequencing and spatial transcriptomics to construct high-resolution expression landscapes of IGFBPs within bone tissues at both single-cell and spatial levels. These approaches will enable the delineation of heterogeneous expression patterns across osteolineage cells, osteoclasts, immune cells, and stromal subpopulations, and facilitate spatial ligand-receptor interaction analyses to uncover region-specific regulatory networks of IGFBPs in local bone remodeling. Animal and cellular experiments have confirmed that IGF-IGFBP can affect the osteogenic and lipogenic differentiation of BMSCs, as well as the differentiation of osteoblast precursor cells. However, in view of the multi-tissue origin of IGFBP and its regulation by hormones or other factors, which makes its relationship with osteoporosis complicated, it is evident that the current studies on IGF-IGFBP family influence on bone metabolism mainly focus on osteogenesis, with little research literature in the direction of lipogenesis and osteoclastogenesis; in osteogenesis, although some IGFBP molecules have been demonstrated to inhibit osteogenesis, no specific mechanistic studies have been addressed, and the results of some studies of the effects of IGFBP on bone metabolism have been inconsistent.

Given that osteoblasts themselves also express IGFBP, the specific mechanisms by which they regulate bone metabolism require further investigation: specifically, whether this regulation depends on extracellular sources of IGFBP or is mediated by IGFBP synthesized by osteoblasts themselves and subsequently altered intracellularly. Furthermore, does a mutual regulatory relationship exist between intracellular and extracellular IGFBP? In the context where multiple IGFBP molecules coexist, how they interact with each other and synergize or antagonize with other hormones to regulate bone metabolism is also a question worthy of attention. As binding proteins for insulin-like growth factors (IGFs), IGFBPs can, on one hand, indirectly influence bone metabolism by modulating the bioavailability of IGFs. On the other hand, they can also act independently of IGFs, directly affecting osteoclast precursor cells or osteoblasts, thereby influencing their differentiation, fusion, and function. Under different physiological or pathological conditions, it remains unclear which of these two mechanisms predominates, or whether they act synergistically in regulating bone metabolism. This uncertainty represents a key question for future research. Additionally, this review has certain limitations. The included studies encompass clinical research, animal experiments, and cell-based assays (Table 1). The levels of evidence from these various study types are inconsistent, but this review did not perform a strict hierarchical grading of evidence. In the several clinical studies included, only correlations were observed between serum IGFBP levels and BMD, osteoporosis, or fracture risk. These findings cannot establish a definitive causal relationship between IGFBP and bone mass. Moreover, no clinical studies have investigated the association between circulating IGFBP levels and those within the bone microenvironment. Future research could focus on this particular direction for further exploration. Undoubtedly, the study of IGFBP affecting bone metabolism is still at an early stage. Currently, there is a lack of experimental therapies or clinical trials targeting the regulation of IGFBP pathways, necessitating more comprehensive and in-depth studies. Since IGFBPs are expressed in multiple tissues and organs, future efforts must also address how to achieve bone-specific therapy while avoiding adverse effects on other tissues. A study has developed a dual-drug delivery system containing PTH and simvastatin, utilizing 3D-printed scaffolds and GelMA hydrogels for bone-specific delivery, demonstrating promising bone regeneration effects in a model of osteoporotic cranial defects. Such delivery strategies could potentially be applied in the future for targeted delivery of IGFBP-related therapies. Furthermore, future research could focus not only on IGFBPs themselves as therapeutic targets but also on their proteolytic enzymes as potential targets for drug development [116]. It is believed that the vitalness of IGFBP studies will increase with the progress of IGFBP cell biology research, and the later use of IGFBP as a biomarker for osteoporosis detection as well as the development of osteoporosis therapeutic approaches targeting the IGFBP pathway are expected.

**Table 1.** Summary of the association between IGF-IGFBP members and bone metabolism.

IGF-IGFBP members	Research subjects	Key quantitative findings	Direction of relevance	Type of research
IGF1	173 elderly men (Average age 64.4 years) and 107 postmenopausal women (Average age 63.6 years)	In women more than 10 years past menopause, IGF1 levels were significantly associated with longitudinal changes in femoral BMD (*n* = 50, *r* = 0.38; *p* = 0.01). In contrast, no such association was observed in women with a menopausal age of 10 years or less (*n* = 39, *r* = 0.15; *p* = 0.36). Serum IGF-1 levels in men were not associated with changes in BMD [38].	Positive relationship in BMD	Human observational data
	5266 osteoporosis patients and 331,893 controls	IGF1 level is associated with a reduced risk of osteoporosis (OR = 0.998, 95% CI = 0.997–1.000, *p* = 0.032) [115].	Negative relationship in osteoporosis	Human observational data
	3203 osteoporosis patients and 209,575 controls	IGF1 were not identified to be associated with osteoporosis [115].	No relationship in osteoporosis	Human observational data
IGF2	344 men and 276 women (ages 21–93)	no associations remained between IGF2(hip *r* = 0.15, spine *r* = 0.12; *p* > 0.05) and BMD after adjustment either for age, bioavailable sex steroids, or muscle mass[49].	No relationship in BMD	Human observational data
IGFBP1	633 men and 506 women (ages 44–98)	IGFBP1 was significantly (*p* < 0.05 for all) associated with BMD at the hip and spine in both men (hip r = −0.141, spine r = −0.149) and women (hip *r* = −0.186, spine *r* = −0.093). After adjusting for age and BMI, there was no significant association between IGFBP1 and BMD at the hip or spine [39].	Negative relationship in BMD	Human observational data
	351 women (ages 69–79)	The relation between IGFBP1 and the risk of a hip fracture was positive and linear, as was the relation between IGFBP1 and the risk of major osteoporotic fractures [40].	Positive relationship in fracture	Human observational data
	OVX mice	In OVX mice, the expression of IGFBP1 in the liver is increased. IGFBP1 contributes to osteoporosis by promoting osteoclast differentiation, and anti-IGFBP1 therapy alleviates OVX-induced osteoporosis [67].	Promotion of bone resorption	Animal models data
IGFBP2	344men and 276 women (ages 21–93)	There was an inverse association of IGFBP2 with BMD (hip *r* = −0.36, spine *r* = −0.24; *p* < 0.01) [49].	Negative relationship in BMD.	Human observational data
	IGFBP2 KO bone marrow cells	Culture of IGFBP2 KO bone marrow cells showed a decrease in the number of osteoclasts and impaired bone resorption function. Adding full-length IGFBP2 restored osteoclast differentiation, fusion, and bone resorption [69].	Promotion of bone resorption	Cells models data
IGFBP3	344men and 276 women (ages 21–93)	No associations remained between IGFBP3 and BMD (hip *r* = 0.11, spine *r* = 0.12; *p* > 0.05)after adjustment either for age, bioavailable sex steroids, or muscle mass [49].	No relationship in BMD	Human observational data
	5266 osteoporosis patients and 331,893 controls	IGFBP3 level is associated with a reduced risk of osteoporosis (OR = 0.999,95% CI = 0.998–1.000, *p* = 0.019)[116].	Negative relationship in osteoporosis	Human observational data
	3203 osteoporosis patients and 209,575 controls	IGFBP3 were not identified to be associated with osteoporosis [116].	No relationship in osteoporosis.	Human observational data
	Transgenic mice overexpressing IGFBP3	Transgenic mice overexpressing IGFBP3 exhibited an increased number of osteoclasts and enhanced bone resorption [51].	Promotion of bone resorption	Animal models data
	Osteoclasts	Addition of IGFBP3 to osteoclast culture medium blocked osteoclast differentiation in a dose-dependent manner [71].	Inhibition of bone resorption	Cells models data
	Adipocytes	Overexpression of IGFBP3 alleviated CHRDL1 silencing-induced attenuation of adipogenic differentiation [54].	Promotion of lipogenesis	Cells models data
IGFBP4	OC-BP4 mice	OC-BP4 mice exhibited targeted overexpression of IGFBP4 in osteoblasts. The number of osteoblasts, bone length, and bone formation rate in OC-BP4 mice were approximately half of those in control mice [56].	Inhibition of bone formation	Animal models data
IGFBP5	OC-BP5 mice	OC-BP5 mice exhibited targeted overexpression of IGFBP5 in osteoblasts. These mice showed a transient reduction in trabecular bone volume, along with decreases in trabecular number and thickness, as well as a transient decrease in the bone mineral apposition rate. Their osteoblasts exhibited reduced expression of ALP, OCN [61].	Inhibition of bone formation	Animal models data
	Osteoclasts	IGFBP5 significantly stimulated bone resorption pit formation by pre-existing mice osteoclasts; however, IGFBP5 did not affect the bone resorption activity of isolated rabbit osteoclasts [73].	Promotion of bone resorption	Cells models data
IGFBP6	MC3T3-E1 cells	Overexpression of IGFBP6 in MC3T3-E1 cells inhibited nodule formation, osteocalcin mRNA expression, and ALP activity^63^].	Inhibition of bone formation	Cells models data

## Data Availability

Data sharing is not applicable to this article as no new data were created or analyzed in this study.
